# Blunted Ambiguity Aversion During Cost-Benefit Decisions in Antisocial Individuals

**DOI:** 10.1038/s41598-017-02149-6

**Published:** 2017-05-17

**Authors:** Joshua W. Buckholtz, Uma Karmarkar, Shengxuan Ye, Grace M. Brennan, Arielle Baskin-Sommers

**Affiliations:** 1 Harvard University, Center for Brain Science, Cambridge, MA 02138 USA; 2 Harvard University, Psychology, Cambridge, MA 02138 USA; 3 Massachusetts General Hospital, Psychiatry, Boston, MA 02114 USA; 4 Harvard Business School, Boston, MA 02163 USA; 5 Yale University, Psychology, New Haven, CT 06520 USA

## Abstract

Antisocial behavior is often assumed to reflect aberrant risk processing. However, many of the most significant forms of antisocial behavior, including crime, reflect the outcomes of decisions made under conditions of ambiguity rather than risk. While risk and ambiguity are formally distinct and experimentally dissociable, little is known about ambiguity sensitivity in individuals who engage in chronic antisocial behavior. We used a financial decision-making task in a high-risk community-based sample to test for associations between sensitivity to ambiguity, antisocial behavior, and arrest history. Sensitivity to ambiguity was lower in individuals who met diagnostic criteria for Antisocial Personality Disorder. Lower ambiguity sensitivity was also associated with higher externalizing (but not psychopathy) scores, and with higher levels of aggression (but not rule-breaking). Finally, blunted sensitivity to ambiguity also predicted a greater frequency of arrests. Together, these data suggest that alterations in cost-benefit decision-making under conditions of ambiguity may promote antisocial behavior.

## Introduction

Antisocial behavior is typified by a chronic pattern of social, legal, and moral norm violations. Canonical manifestations of antisocial behavior include physical aggression, substance abuse, and other forms of criminal offending. Engaging in these proscribed acts is inherently “risky,” as doing so carries a non-zero probability of adverse outcomes. In the case of aggression and criminal behavior, such outcomes could include arrest and incarceration (i.e. formal third-party sanction by the state) as well as physical harm by the target of the antisocial act (i.e. informal second-party retaliation). Given the strong association between risk-taking and antisocial behavior, some suggest that insensitivity to risk could predispose the development of antisocial syndromes^1^.

Several lines of research provide empirical support for the notion of diminished risk processing in antisocial individuals. Epidemiological studies show that risk preferences and risk-taking traits are positively associated with the frequency and severity of delinquency, violence, and substance abuse in both adolescents and adults^2^. Accordingly, antisocial individuals perform sub-optimally on experimental paradigms that require selection among actions with probabilistic reward outcomes^3–5^. Data from longitudinal and genetically informed developmental designs suggest that heritable individual differences in risk sensitivity exert a causal influence on antisocial behavior^6, 7^. Together, work to date appears to show that antisocial individuals are relatively insensitive to risky prospects during decision-making.

The link between antisocial behavior and risk sensitivity is noteworthy given that antisocial behavior is present in other behavioral disorders characterized by impaired risk processing, such as problem gambling and substance abuse^7, 8^. These associations appear to be driven by a common genetic diathesis^9^, suggesting shared biological and cognitive mechanisms^9, 10^. Consistent with this, problem gamblers, substance abusers, and antisocial individuals show a similar pattern of aberrant risk processing^11–13^. On the whole, these findings provide support for the hypothesis that a trait-like deficit in risk sensitivity predisposes a dimension of psychopathology that includes antisocial behavior.

While the work noted above suggest that antisocial individuals both engage in risky behavior and show risk processing deficits in laboratory tasks, the specific processes at issue remain somewhat underspecified. Drawing from the economics literature allows us to refine the definition of “risky”, and to formalize an important distinction between risk and ambiguity. In decisions under risk, the outcomes of one’s choices have known (or knowable) probabilities. By contrast, deciding under ambiguity entails making a choice when the outcome probabilities are unknown, and undiscoverable through logical deduction or inductive inference^14, 15^. As with risk, people are, on average, ambiguity-averse^16–19^. Moreover, risk and ambiguity preferences are dissociable^16–19^, with distinct neural mechanisms^20^ and selective associations to psychopathology^20, 21^.

Considered within the framework described above, the “risky” decisions of antisocial individuals may more accurately be described as “ambiguous.” The precise outcome probabilities of committing a crime are unknown and unknowable. For example, an individual deciding whether or not to mug any of 10 people on a street doesn’t know the likelihood of success (getting away with it) or failure (getting caught) for any of those 10 potential victims. Nor can they ascertain the exact probability of being incarcerated for a given length of time if convicted. To date, however, the role of ambiguity preferences in predisposing antisocial behavior remains unknown.

Their strong propensity towards maladaptive decision-making under conditions of uncertainty, especially in the context of reward pursuit, suggests that antisocial individuals are relatively insensitive to ambiguity. In the present study, we tested this hypothesis using a financial choice paradigm^22^ in a high-risk community sample. Economic models of risk and ambiguity were fit to participants’ data; the resulting risk and ambiguity parameters were examined for broad and subtype-specific associations with clinical and real-world measures of antisocial behavior. We predicted that ambiguity sensitivity would be lower in individuals meeting diagnostic criteria for antisocial syndromes and in those who have been arrested more frequently.

## Methods and Materials

### Participants

We used a targeted recruitment approach in a high-crime community to enrich our sample for antisocial behavior. 62 male (80.5%) and 15 female (19.5%) adults aged 18 to 55 (*M* = 36.74, *SD* = 11.65) were recruited from the community through flyers calling for individuals who engage in risk-taking behavior (e.g., crime, substance use, gambling, impulsive behavior, bullying) in New Haven County, Connecticut, a high-crime region. New Haven ranks in the 94^Th^ percentile for crime; on average, 358 crime are committed per square mile, as compared to the national median of 32.8 (Note: Data accessed from http://www.neighborhoodscout.com/ct/new-haven/crime/ on 06/01/2016.). The rate of violent crime is 10.62 (per 10,000 residents), compared to a statewide rate of 2.37 and a national median of 3.8. This demographic feature, combined with our targeted recruitment of self-identified “risk-takers” resulted in a sample that was enriched for clinically significant antisocial behavior (see Results).

A prescreen phone interview and in person assessment materials were used to exclude individuals who were younger than 18 or over 55, had performed below the fourth-grade level on a standardized measure of reading (Wide Range Achievement Test-III)^23^, who scored below 70 on a brief measure of IQ^24^, who had diagnoses of schizophrenia, bipolar disorder, or psychosis, not otherwise specified^25^, or who had a history of medical problems (e.g., uncorrectable auditory or visual deficits; head injury with loss of consciousness greater than 30 minutes) that may impact their comprehension of the materials or performance on the task. All participants provided written informed consent and all experimental procedures were completed in line with the protocol approved by the Yale University Human Investigation Committee. Participants earned $10/hour for their completion of the self-report measures and the experimental task.

The majority of participants self-identified as Black/African American (37.7%) or as White (36.4%), with the remainder of the sample identifying as mixed racial identity (6.5%) or Asian (1.3%). Almost half of participants in the sample (45.5%) were unemployed, while the remainder were employed either full-time or part-time (37.7%), full-time students (11.7%), or on disability (5.2%). Educational attainment was as follows: 31.2% high school diploma, GED, or less; 45.5% vocational school, some college or Bachelor’s degree; and 5.2% graduate work or degree.

All participants were asked if they were ever arrested; if affirmative, participants provided the number of times they’d been arrested. This self-report was confirmed using the State of Connecticut Department of Correction inmate database. Approximately, sixty-five percent of the sample had been arrested prior to participation in the study. The number of arrests ranged from 0–40 (mean = 3.64, SD = 6.26).

### Clinical Measures

#### Externalizing Spectrum Inventory-Brief (ESI)

The ESI-Brief is a 100-item self-report questionnaire that assesses^26^ a range of behavioral and personality characteristics associated with the externalizing spectrum of psychopathology. This version was derived from Krueger, Markon, Patrick, Benning, and Kramer’s (2007) 415-item self-report measure and is correlated *r* = 0.98 with the original measure^27^. The items consist of statements regarding specific behaviors and qualities, and participants are asked to choose the response that best describes them on a 4-point Likert scale: True (1), Mostly True (2), Mostly False (3), or False (4). Prior to scoring, the appropriate items were reverse coded. For the present sample, the internal consistency (Cronbach’s alpha) was 0.981.

#### Subtypes of Antisocial Behavior Questionnaire (STAB)

The Subtypes of Antisocial^28^ Behavior Questionnaire (STAB) is a 32-item self-report measure that yields a total score (STAB total), as well as evaluates three distinct subtypes of antisocial behavior: physical aggression (STAB-PA; “Got into physical fights”), rule-breaking (STAB-RB; “Shoplifted things”), and social aggression (STAB-SA; “Tried to hurt someone’s feelings”). Participants are asked to respond based on a 5-point Likert scale (1 = never; 2 = hardly ever; 3 = sometimes; 4 = frequently; 5 = nearly all the time). For the present sample, the internal consistency (Cronbach’s alpha) was 0.847.

#### Self-Report Psychopathy scale, version III (SRP-III)

The Self-Report Psychopathy Scale (Paulhus, Hemphill & Hare, in press﻿^29^) is a 64-item measure looking at four subscales of psychopathic behavior: Interpersonal Manipulation (IPM), Callous Affect (CA), Erratic Life Style (ELS), and Anti-Social Behavior (ASB). Participants are asked to rate the degree to which they agree with each statement using a 5-point Likert scale, where 1 means “Disagree Strongly” and 5 means “Agree Strongly”. To score, the 16 items in each subscale are averaged to get the mean. The overall SRP-III score is the mean of the four subscales. A higher score indicates a greater level of psychopathic behavior. For the present sample, the internal consistency (Cronbach’s alpha) was 0.781.

#### Structured Clinical Interview for DSM-IV Disorders (SCID-IV)

The SCID-IV was used to determine^25^ Antisocial Personality Disorder (APD) diagnosis. A diagnosis of APD was given if there was evidence of Conduct Disorder prior to age 15 and sufficient adult antisocial symptoms. The prevalence of APD is typically 3% in the general population; however, APD and antisocial behavior are more prevalent among individuals with low socioeconomic status and in residents of high crime neighborhoods^30^. Inter-rater reliability for APD based on 25% of the sample was 0.99.

### Ambiguity Decision-Making Task

**Figure 1:**
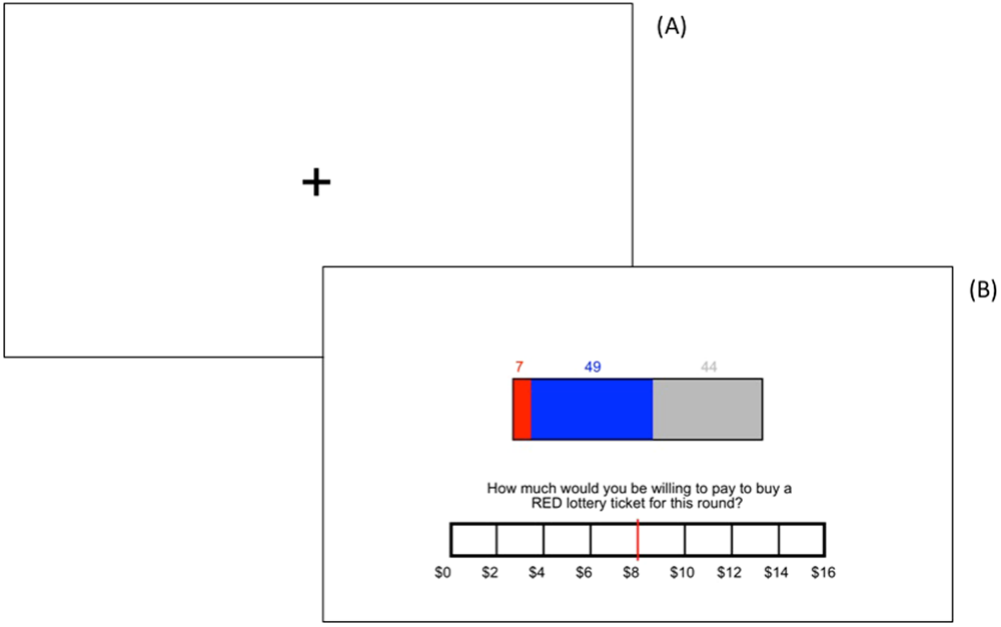
Ambiguity Task Diagram. After a brief fixation period (**A**), participants were presented with a virtual “bag” of exactly 100 poker chips, all of which are colored either red or blue. On each trial, participants receive partial information about the number of red (winning) and blue (losing) chips. Participants were asked to indicate their willingness to pay (WTP) for a red ticket (**B**). In this sample trial, there are 7 red (winning) chips visible, 49 blue (losing) chips visible, and 44 chips that are not visible and thus not known to the participant. Participants selected a response to the question by moving a marker across the response bar.

Participants completed a computerized decision-making paradigm in which the amounts of favorable and unfavorable information regarding an ambiguous financial prospect were parametrically manipulated^15, 22^. On each trial, participants were presented with a (distinct) virtual “bag” of exactly 100 poker chips, all of which were colored either red or blue. Participants were asked to indicate their willingness to pay (WTP) for a “red” ticket to play a game in which a single chip is drawn from the bag. If the selected chip is red, they win $30, if it is blue, they win nothing (and would lose the ticket price.) While making this choice, participants receive partial information about the number of red and blue chips in the bag (Fig. 1). Parametrically varying the number of red chips and blue chips shown to participants allows for a trial-wise calibration of the availability of favorable or unfavorable information. Since red is the “winning” color, red chips represent favorable information in the context of the task, whereas blue chips represent unfavorable information. The task consisted of 45 trials. Each trial began with a fixation cross, after which participants viewed the available information about the bag’s contents. Participants rated their WTP for a red lottery ticket for the current round (range: $0–16) by sliding a marker across a rating bar displayed at the bottom of the screen. Subjects were not placed under time constraints for responding.

A regression model was used to estimate individual participant sensitivity to favorable information and unfavorable information. Separately for each subject, WTP for each trial was regressed on the number of red and the number of blue chips revealed in that trial. Thus, since selection of a red chip indicates a desired outcome, the coefficient for “Red” indicates the relative influence of favorable information on WTP decisions (i.e., the increase in WTP for every increase in one red chip). Conversely, the coefficient for “Blue” indicates the incremental impact of unfavorable information on WTP.

We adapted a widely used modeling approach to calculate individual estimates of risk and ambiguity sensitivity. For the ambiguous prospect represented on any one trial, we can define *x* as the prize amount ($30) in the task, *p* as the subjective probability of winning, and *A* as the objective amount of ambiguity, which can be normalized between 0 (p is objectively known) and 1 (no information about probability is offered.). We can then use WTP to represent utility (*U*) in the following function (a simplified version of the general model in Gilboa & Schmeidler, 1989) expressing the evaluations of ambiguous prospects in our task^31^.

1 $$U=WTP=(p-\lambda Ap){x}^{\alpha }$$

In this equation, λ is an individually defined scaling factor that captures the subjective sensitivity to objective ambiguity. As such, λ = 0 represents an ambiguity-neutral attitude, while λ > 0 is associated with ambiguity aversion or sensitivity, and λ < 0 is associated with an ambiguity-seeking attitude. On the other hand, α denotes an individual’s risk sensitivity, such that *α* = 1 represents a risk-neutral attitude, while *α* > 1 corresponds with risk-seeking, and *α* < 1 corresponds with risk aversion.

We can then estimate a more refined model of the participants’ decision-making by considering the participants’ decision process as a function of utility (as described above) versus costs (which are scaled by α, mirroring the scaling of the reward x in the utility equation):

2 $$\mathrm{Likelihood}\,\mathrm{of}\,\mathrm{Purchase}=lP=\frac{{e}^{{\gamma }^{U}}}{{e}^{{\gamma }^{U}}+{e}^{{\gamma }^{\cos {t}^{\alpha }}}}$$

Note that γ reflects a “randomness” parameter used to reflect the overall degree of how much costs and benefits impact the purchase. We estimate our parameters of interest (*α*, *λ*) by optimizing over the following likelihood function (with WTP normalized between 1 and 0):

3 $$\sum {({\rm{l}}{\rm{o}}{\rm{g}}lP-{\rm{l}}{\rm{o}}{\rm{g}}\frac{WTP}{8})}^{2}$$

### Effect of Favorable/Unfavorable Information on WTP

We used a linear mixed model in STATA 14 to determine the influence of favorable and unfavorable information on WTP decisions. The number of red chips (favorable) and the number of blue chips (unfavorable) were used as fixed effect predictors of WTP; subject was included as a random effect. Age and sex were included as covariates.

### Outlier Detection and Power Analysis

We defined upper and lower bounds for including data as follows: upper bound = 75^th^ percentile + 1.5*Inter-quartile Range, lower bound = 25^th^ percentile − 1.5*Inter-quartile Range. Values that were below the lower bound and above the upper bound were considered univariate outliers and excluded from further analysis. Participants with no behavioral variability (i.e. identical WTP responses for every trial) were also excluded. We also conducted a power analysis to ensure that we were sufficiently powered to obtain statistically significant effects.

### Individual Difference Analyses

We used robust regression in STATA14 to assess relationships between favorable/unfavorable influence parameters (Beta_Red_, Beta_Blue_, Beta_Red_–Beta_Blue_), model-based risk and ambiguity parameters (λ and *α*) and clinical and trait measures. Also, a negative binomial regression model was used to assess the relationships among arrests and ambiguity sensitivity and risk sensitivity. Age and sex were included as covariates in all analyses.

## Results

### Participant Characteristics

Task data were collected for 77 participants. Three participants were excluded because they exhibited no behavioral variability, leaving 74 participants for analysis (mean age: 36.59, SD = 11.74, range = 18–55; 59 male and 15 female). Within this sample, 22 participants met criteria for APD. ESI total scores ranged from 100–355 (mean = 192.34, SD = 64.84). SRP-III total scores ranged from 86–236 (mean = 156.73, SD = 30.68). STAB total scores ranged from 32–121 (mean = 67.12, SD = 19.84). Supplementary Table 1 includes additional information about psychiatric diagnoses present in this sample.

### Data Quality Control

We detected 1 outlier Beta_Red_ value, 5 outlier Beta_Blue_ values, 15 outlier α values, and 13 outlier *λ* values. Outlier values were not included in individual difference analyses, leaving 73 participants for Beta_Red_ analyses, 69 participants for Beta_Blue_ analyses, 59 participants for *α* analyses, and 61 participants for *λ* analyses (see Supplementary Material). Demographic and psychiatric variables (e.g. age, sex, ESI total scores, SRP-III total scores, and STAB total scores) did not differ between excluded and included participants for any of the analyses (all p-values > 0.2). Power analyses indicated that after removing the relevant outliers all models provided sufficient power, at 87% to 94%, to detect a moderate effect size (d = 0.50 with an alpha of 0.05). Notably, all results reported as significant remain significant in the full sample (i.e. including those individuals identified as outliers; see Supplementary Results).

### Effects of Favorable and Unfavorable Information on WTP

Across participants, the favorable information (e.g. number of red chips) and unfavorable information (e.g. number of blue chips) shown on each trial were significant and independent predictors of willingness to purchase decisions (Beta_Red = _0.06, 95% CI = 0.051–0.071, z = 12.17; Beta_Blue = _−0.02, 95% CI = −0.025 to −0.013, z = −5.91; both p-values < 0.001). There was no relationship between the coefficients on favorable and unfavorable information (e.g. Beta_Blue_ and Beta_Red_; p = 0.27). Prior work on decision-making under ambiguity suggests that individuals show a bias towards favorable information under conditions of uncertainty^22^. To test for such a bias in the current sample, we subtracted each participant’s Beta_Blue_ values from their Beta_Red_ values to create a favorability bias score for each subject (mean = 0.76, SD = 0.38, range = −0.23–1.27). A one-sample t-test on this value was significant (t = 16.40, p < 0.001), confirming that on average, favorable information had a stronger influence on WTP decisions compared to unfavorable information, replicating prior work using this paradigm^22^.

### Risk and Ambiguity Parameters

**Figure 2:**
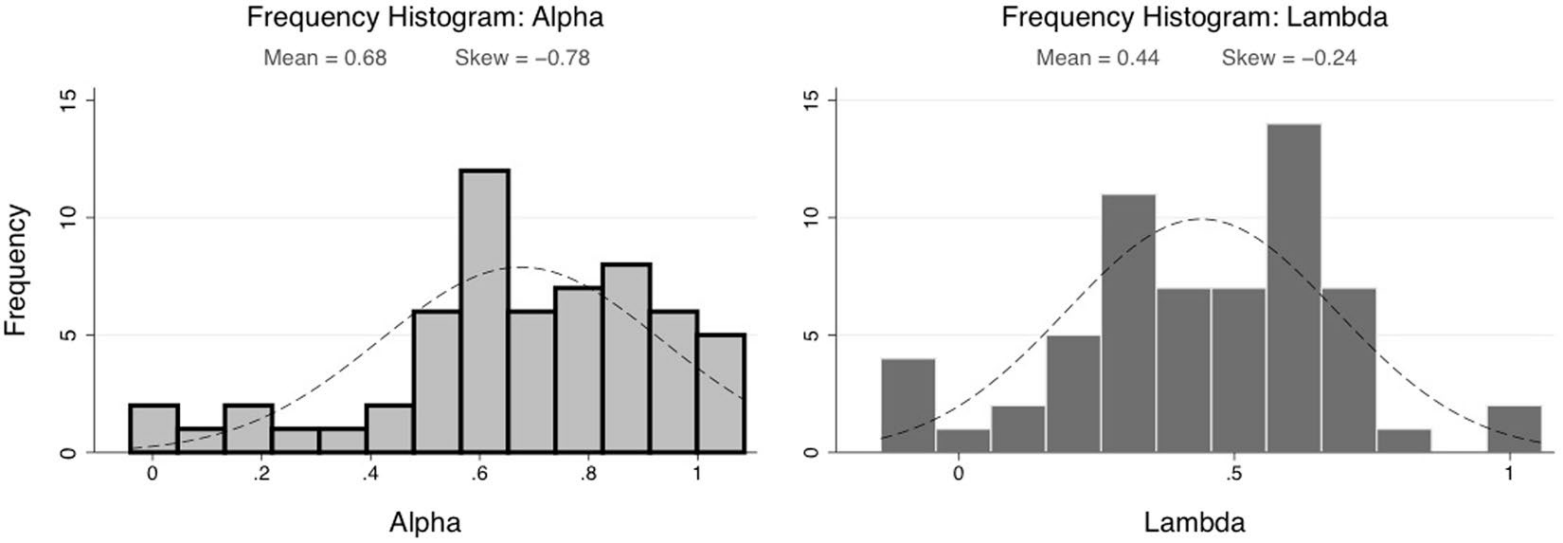
Frequency Histograms for Risk and Ambiguity Parameters. Histograms depict the distribution of alpha (left) and lambda (right) values within this sample. Normal distribution fit is shown with dashed line.

We fit each participant’s choice data to a model of risk and ambiguity sensitivity (see Methods for model details). Our model yielded two parameters, *α* and *λ*, corresponding to risk and ambiguity (lower *α* = more risk averse, higher *λ* = more ambiguity averse). One-sample t-tests confirmed that mean *α* values were significantly less than 1 (t = −9.65, p < 0.001, test value = 1), and that mean *λ* values were significantly greater than 0 (t = 14.03, p < 0.001, test value = 0). Most subjects exhibited aversion to both risk (mean *α* = 0.68, SD = 0.26, range = −0.04–1.09, skew = −0.81) and ambiguity [mean *λ* = 0.44, SD = 0.24, range = −0.14–1.04, skew = −0.25). Of note, considerable inter-individual variability was observed for both parameters (Fig. 2).

In this sample, risk sensitivity (α) and ambiguity sensitivity (λ) values were positively correlated (r^2^ = 0.20, p < 0.001). In addition, α values were positively associated with individual coefficients for favorable, but not unfavorable information (Beta_Red_: r^2 = ^0.11, p = 0.01; Beta_Blue_: p = 0.43). In contrast, λ values were positively associated with coefficients for unfavorable, but not favorable information (Beta_Blue_: r^2 = ^0.13, p = 0.006; Beta_Red_: p = 0.17).

### Individual Difference Analyses

#### Antisocial Personality Disorder

**Figure 3:**
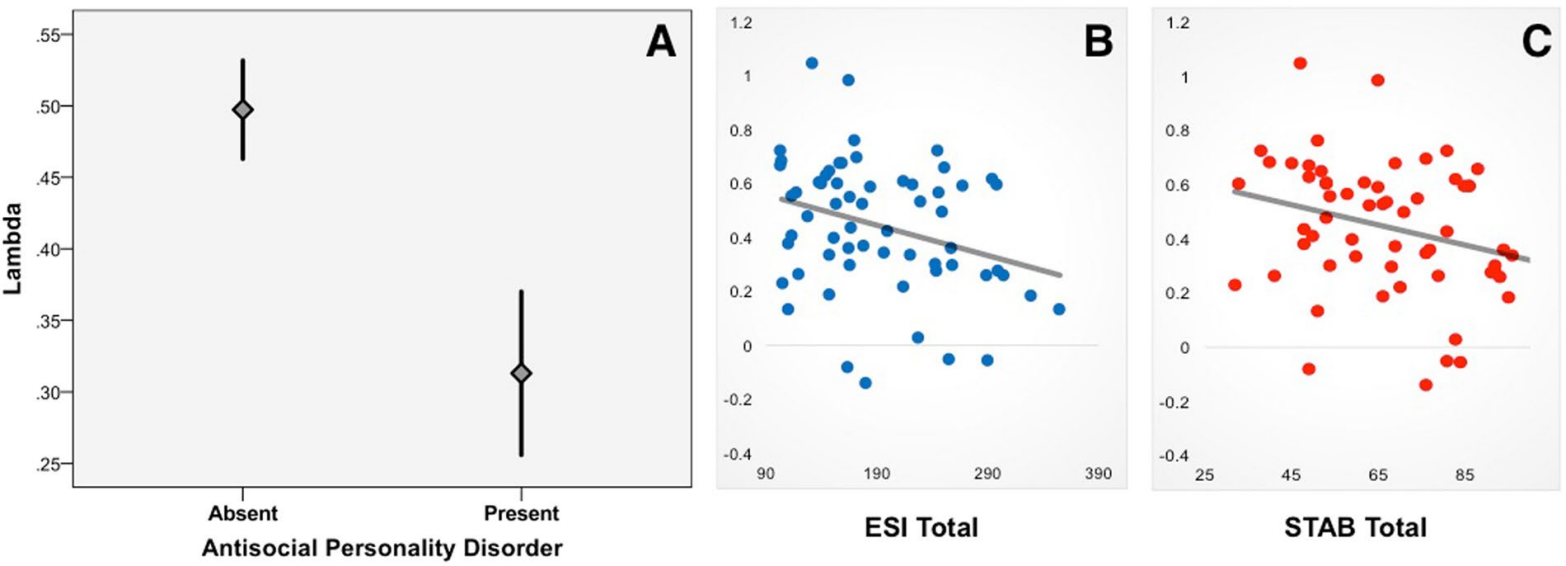
APD Diagnosis and Antisocial Traits are Linked to Blunted Ambiguity Aversion. Scatterplots show lower ambiguity aversion in APD (A; mean +/− 1 SEM), and negative correlations between ambiguity aversion and ESI (**B**) and STAB (**C**) scores.

We did not observe any significant associations between APD diagnosis and Beta_Red_ (p = 0.13), Beta_Blue_ (p = 0.88), or Beta_Red_–Beta_Blue_ (p = 0.60) values. *α* values were not associated with APD diagnosis (p = 0.89); however, *λ* values were significantly lower in participants who met criteria for APD (p < 0.01; mean difference = 0.18; Fig. 3a). The association between ambiguity sensitivity and APD diagnosis remained significant even after controlling for risk sensitivity (i.e. by including *α* values as a predictor in the regression model; p < 0.05). Thus, participants with APD showed reduced ambiguity aversion during cost-benefit decision-making. However, the DSM-IV diagnosis of APD is a broadband measure that is non-selective for the varied subtypes of antisocial behavior. In fact, there is considerable heterogeneity within this broad psychiatric taxon. Empirical data provide strong support for two fractionations - externalizing vs. psychopathy and aggression vs. rule-breaking. We next examined subtype-specific associations to risk and ambiguity preferences^32–34^.

#### Externalizing and Psychopathy

Externalizing reflects a symptomatically unified and etiologically coherent dimension of antisocial behavior, chiefly characterized by disinhibition (e.g. impulsivity) and negative affect (e.g. reactive aggression)^27^. By contrast, psychopathy encompasses aspects of social and emotional dysfunction that distinguishes it from externalizing. Interpersonal (e.g. pathological lying and manipulation) and affective (callousness, diminished empathy) deficits are considered central distinguishing symptoms^35, 36^. We used the Externalizing Symptom Inventory (ESI) to measure externalizing; psychopathy was assesed using the Self-Report Psychopathy Scale-III (SRP-III; see Methods for details on assessment instruments). To identify subtype-specific associations with risk and ambiguity sensitivity, we first examined a series of independent regression models that separately modeled the effects of ESI and SRP-III scores on α and λ values. ESI total scores were not significantly associated with ***α*** values (p = 0.83); however, we observed a significant negative relationship between ESI total scores and ***λ*** values (B = −0.001, SD = 0.005, 95% CI = −0.002 to −0.0003 t = −2.54, p = 0.01). The association between ambiguity sensitivity and externalizing remained significant even after controlling for risk (***α*** values; p < 0.05). By contrast, SRP-III total scores were not significantly associated with either ***α*** (p = 0.25) or ***λ*** (p = 0.13) values.

Second, we used a simultaneous regression approach to determine whether the findings above held after controlling for the common variance between ESI and SRP-III scores. ESI total scores were not significantly associated with ***α*** values (p = 0.463); however, we observed a significant negative relationship between ESI total scores and *λ* values (B = −0.002, SD = 0.001, 95% CI = −0.002 to −0.0003 t = −2.54, p = 0.042). The association between ambiguity sensitivity and externalizing remained significant even after controlling for risk (***α*** values; p < 0.05). SRP-III total scores were not significantly associated with either ***α*** (p = 0.217) or ***λ*** (p = 0.651) values. Taken together, these results suggest that individuals with high levels of externalizing, but not psychopathy, have reduced ambiguity aversion (Fig. 3b). However, risk preferences during decision-making under uncertainty did not vary as a function of either externalizing or psychopathy.

#### Rule-Breaking and Aggression

Developmental, epidemiological and genetic studies highlight two etiologically and clinically distinct subtypes of antisocial behavior: rule breaking (e.g. lying, covert theft, destruction of property) and aggression (e.g. physical attacks against others, bullying). *α* values were not significantly associated with STAB total, STAB-RB, STAB-PA or STAB-SA scores. Higher STAB total scores predicted lower *λ* values (B = **−**0.004, SD = 0.002, 95% CI = **−**0.008 to **−**0.0005, t = **−**2.26, p = 0.03). This association appeared to be driven by the STAB-PA subscale (B = **−**0.012, SD = 0.005, 95% CI = **−**0.023 to **−**0.001, t = **−**2.23, p = 0.03; p-values for STAB-SA and STAB-RB were 0.513 and 0.722 respectively). The association between ambiguity sensitivity and aggression remained significant even after controlling for risk (***α*** values; p < 0.05). These results suggest that blunted ambiguity aversion is selective for aggressive, but not rule breaking, forms of antisocial behavior (Fig. 3c).

#### Arrest Record

The results above show that relatively lower ambiguity aversion is linked to clinically significant levels of impulsive-aggressive antisocial behavior. Using participant arrest history, we assessed the relevance of ambiguity aversion for real-world antisocial behavior. Participants with lower ***λ*** values were arrested more frequently than those with higher ambiguity aversion (B = −2.09, SE = 0.90, z = −2.31, p = 0.021, 95% C.I.: −3.86 to −0.32). The use of a zero-inflated negative binomial model to account for the relatively high frequency of zero arrest values revealed similar results (z = −2.39, p = 0.017).

## Discussion

Replicating prior work, we show that people are – on average – ambiguity averse during cost-benefit decision-making under conditions of uncertainty, and observed substantial individual variability in ambiguity aversion. At the level of broad diagnosis, subjects with APD showed significantly less sensitivity to ambiguity compared to participants that did not meet criteria for the disorder. Additionally, we found evidence that the association between antisocial behavior and ambiguity aversion was subtype-specific, being evident for impulsive-aggressive participants but not for those high in psychopathy and rule-breaking. Blunted ambiguity aversion was also a significant predictor of arrest frequency, suggesting real-world behavioral correlates for ambiguity preferences expressed in financial situations.

Many have noted that APD diagnostic criteria don’t distinguish between several putative subtypes of antisocial behavior^37^. While a consensus account of the taxonomy of antisocial behavior remains to be developed, there is broad agreement regarding two phenotypic distinctions. The first fractionates antisocial behavior into externalizing and psychopathy, which differ according to the absence (in the former) or presence (in the latter) of interpersonal-affective deficits (e.g. callousness)^27, 35, 36^. The second distinction is made on the basis of divergent behavioral manifestations of antisocial behavior: rule-breaking vs. aggression^38^. The current data show that diminished ambiguity aversion is evident for externalizing and for aggressive antisocial traits, but not for psychopathy or rule-breaking. Such dimensional selectivity provides additional behavioral evidence for the existence of these subtypes, and supports the notion that dimension-specific cognitive and neurobiological mechanisms can predispose a common behavioral endpoint (antisocial behavior)^34, 39^.

The current findings support a recent and growing body of work linking ambiguity preferences to psychopathology. For example, Pushkarskaya and colleagues (2015) reported that patients with obsessive-compulsive disorder exhibited heightened aversion to ambiguity – but not risk – during cost-benefit decision-making^40^. Tymula and colleagues (2012) found that adolescent participants showed reduced ambiguity aversion compared to adults. Further, across adolescent subjects, lower sensitivity to ambiguity predicted higher scores on a measure of behavioral “recklessness”^41^. Likewise, in a sample of adult problem gamblers, weaker ambiguity, but not risk, aversion was found to predict higher levels of dysfunction and impairment^42^. Our finding of decreased ambiguity sensitivity during cost-benefit decision-making in impulsive-aggressive antisocial individuals is highly consistent with these data.

Moreover, this study supports a growing literature linking aberrant value-based decision-making to antisocial behavior. Whereas cognitive and neurobiological models of antisociality have largely focused on deficient inhibitory control (i.e. response inhibition) as a mechanism for criminal behavior and substance abuse, more recent work suggests that aberrant value representation and cost-benefit integration may play a prominent role in predisposing antisocial behavior^43, 44^. These data accord well with prior work implicating dysfunctional reward and motivation-related processes as a substrate for impulsivity, aggression, and antisociality^1, 45^ and further highlight the utility of examining value-based decision-making in these populations.

While significant associations were observed for ambiguity sensitivity, we did not find evidence of a link between antisocial behavior and risk preferences in our task. On its face, this finding is somewhat surprising, given the wealth of evidence linking antisocial behavior to risk-related traits and behaviors^1–3^. There are two potential explanations for our failure to detect this association. One possibility is that antisocial individuals are not, strictly speaking, insensitive to *risk*. In other words, previously observed “risk” biases in antisocial individuals may have been confounded by occult differences in ambiguity sensitivity. As has been detailed elsewhere^14, 18, 19^, risk and ambiguity are conflated in the design of several frequently used “risky decision-making” tasks (e.g. the Iowa Gambling Task and Balloon Analog Risk Task). This limitation makes it difficult to ascribe variability in task performance to risk sensitivity, *per se*. The current modeling approach allows for a formal dissociation of variance in choice behavior attributable to risk vs. ambiguity sensitivity in the general context of ambiguous decision-making. It is possible that our ability to separate the relative contributions of risk and ambiguity in uncertain situations may account for the discrepancy between the current results and prior findings of risk insensitivity in antisocial behavior.

Alternatively, the present failure to detect an association between risk preferences and antisocial behavior may reflect the specific design of our ambiguity decision-making paradigm. While risk sensitivity can be modeled with the current task, the design was optimized to measure ambiguity. In particular, the number of “risky” lotteries (e.g. 50 red chips or 50 blue chips) is small compared to the number of ambiguous ones. Thus risk sensitivities are generally estimated as the response to the risk component of an ambiguous decision. This question could be resolved in future work that assesses the concordance of α values derived from the current task with a measure of “pure” risk sensitivity (e.g. probability discount rate)^46^.

Several limitations to this study are worth noting. First, we used a sample of community volunteers rather than incarcerated offenders. Such samples are often limited in the range of antisocial behavior exhibited by participants, raising doubts about the validity and generalizability of inferences. This does not appear to be an issue for the current sample, which was relatively enriched for antisocial behavior: >25% of the sample met criteria for APD, and there was significant variability in real-world criminal behavior. While this is, at first glance, a perhaps surprisingly high rate of offending for a community sample, New Haven C.T. ranks in the 94^Th^ percentile for crime among American cities of comparable size: on average, 358 crime are committed per square mile, as compared to the national median of 32.8 (Note: Data accessed from http://www.neighborhoodscout.com/ct/new-haven/crime/on 06/01/2016.). The use of a targeted recruitment approach in a crime-enriched community likely accounts for the high rate of antisocial behavior observed within this sample. Second, it is worth considering whether the hypothetical nature of the ambiguous financial prospects in the current task limits interpretation of the current findings. Previous research employing both hypothetical and incentive compatible versions of the task has demonstrated that people show comparable patterns of behavior, regardless of the specific incentive structure^22^. In addition, while real financial stakes might influence the absolute magnitude of participants’ value estimates, it is less likely to shift the *relative* sensitivity to the levels of information in the task, which is at the heart of these findings. Nevertheless, future work in this area should endeavor to replicate the present findings in an incarcerated sample using an incentive-compatible version of the task.

Decision-making under ambiguity defines our choice landscape. Whether we’re deciding what to order on a restaurant menu, how many cocktails to consume at a bar, or which car to steal, the outcomes of our choices rarely have knowable probabilities. This work links inter-individual variation in ambiguity sensitivity to clinical and real-world measures of antisocial behavior. Of course, this single aspect of decision-making is likely neither necessary nor sufficient to produce aggression, substance abuse and criminal offending. Rather, antisocial behavior undoubtedly arises from multiple deficits in diverse domains of cognition (e.g., social norm learning, norm representation, forward action-outcome mapping, empathy, etc.), which exert additive or interactive effects on risk for antisocial syndromes. However, while the risk architecture of antisocial behavior is clearly multifactorial, the current data strongly implicates cost-benefit decision-making generally – and ambiguity preferences specifically – as an important and understudied susceptibility factor.

## Electronic supplementary material

Supplementary Material
