# Supplementary material for: Blunted Ambiguity Aversion During Cost-Benefit Decisions in Antisocial Individuals
Source: Sci Rep. 2017 May 17;7:2030. doi: 10.1038/s41598-017-02149-6 (PMC5435701; doi:10.1038/s41598-017-02149-6)
Supplement: Supplementary file 1 — Supplementary Material [file 41598_2017_2149_MOESM1_ESM.docx]

**Supplementary Material**

Blunted Ambiguity Aversion During Cost-Benefit Decisions in Antisocial Individuals

­­

Joshua W. Buckholtz, Ph.D.^1,2,3*^, Uma Karmarkar, Ph.D.^1,4^, Shengxuan Ye, M.Sc.,^4^, Grace M. Brennan, B.A.^5^ and Arielle Baskin-Sommers, Ph.D.^5*^

^1^ Harvard University, Center for Brain Science (Cambridge, MA, 02138)

^2^ Harvard University, Psychology (Cambridge, MA, 02138)

^3^ Massachusetts General Hospital, Psychiatry (Boston, MA, 02114)

^4^ Harvard Business School (Boston, MA, 02163)

^5^ Yale University, Psychology (New Haven, CT, 06520)

*These authors contributed equally to this work

Correspondence to:

Joshua Buckholtz

Northwest Building, East Wing

Room 295.01

52 Oxford St.

Cambridge, MA 02138

T 617-496-8588

F 617-496-8607

[joshua_buckholtz@harvard.edu](mailto:joshua_buckholtz@harvard.edu)

Arielle Baskin-Sommers

2 Hillhouse

New Haven, CT 06511

T 203-432-9257

arielle.baskin-sommers@yale.edu

SUPPLEMENTAL METHODS

Supplementary Table 1. Psychiatric Diagnoses Present in Current Sample.

| Diagnosis | # Meeting Criteria |
| --- | --- |
| Major Depression Current/Past | 0/13 |
| Dysthymia | 1 |
| Schizophrenia | 0 |
| Bipolar Disorder | 0 |
| Alcohol Abuse/Dependence | 22/16 |
| Cannabis Abuse/Dependence | 21/9 |
| Stimulant Abuse/Dependence | 2/3 |
| Opioid Abuse/Dependence | 0/6 |
| Cocaine Abuse/Dependence | 3/8 |
| Poly Drug Dependence | 6 |
| Panic Disorder | 1 |
| Social Phobia | 2 |
| Specific Phobia | 2 |
| Obsessive-Compulsive Disorder | 1 |
| Post-Traumatic Stress Disorder | 1 |
| Generalized Anxiety Disorder | 0 |
| Narcissistic Personality Disorder | 3 |
| Borderline Personality Disorder | 6 |
| Antisocial Personality Disorder | 22 |
| Conduct Disorder (Past) | 24 |

SUPPLEMENTAL RESULTS

Consideration of outliers

In our initial sample of 77 participants, three were dropped due to the absence of behavioral variability. Of the remaining 74, we identified a number of extreme outliers in our model-based alpha (risk sensitivity; 15 outliers – 20%) and lambda (ambiguity sensitivity; 17.5%) parameters. Given the number of outliers, the concern is that the results are not robust; i.e. the finding of decreased ambiguity aversion in antisocial behavior is an artifact of outlier removal. To address this concern we ran a series of control analysis which together show that these results are in fact quite robust.

First, we re-ran our robust regression analyses with the full sample (n=74). Significant negative associations with ambiguity sensitivity and APD diagnosis (p=0.003), APD symptom severity (p=0.025), Externalizing Symptom Inventory (ESI) total score (p=0.016), and Subtypes of Antisocial Behavior (STAB) total score (p=0.015). No associations were observed between any of these measures and risk sensitivity (all p-values >0.65). These data show that we observe reduced ambiguity sensitivity in antisocial behavior regardless of whether or not we remove outlier data points.

Second, we ran a series of complimentary mixed-effects regression analysis, using Willingness to Pay as the dependent measure. We included trial-wise ambiguity (# of gray chips shown), antisocial measures, and ambiguity-antisocial measure interactions as predictors; participant was treated as a random effect. Consistent with our model-based analyses of alpha and lambda, we observed significant interactions between our measures of antisocial behavior and trialwise variation in ambiguity (p<0.001), such that willingness to pay decreased sharply with increasing ambiguity for low antisocial individuals; however, high antisocial individuals appeared relatively insensitive to the effect of ambiguity on choice behavior (SFig. 1). These data further indicate the robustness of our findings to both outlier exclusion and analytical approach.


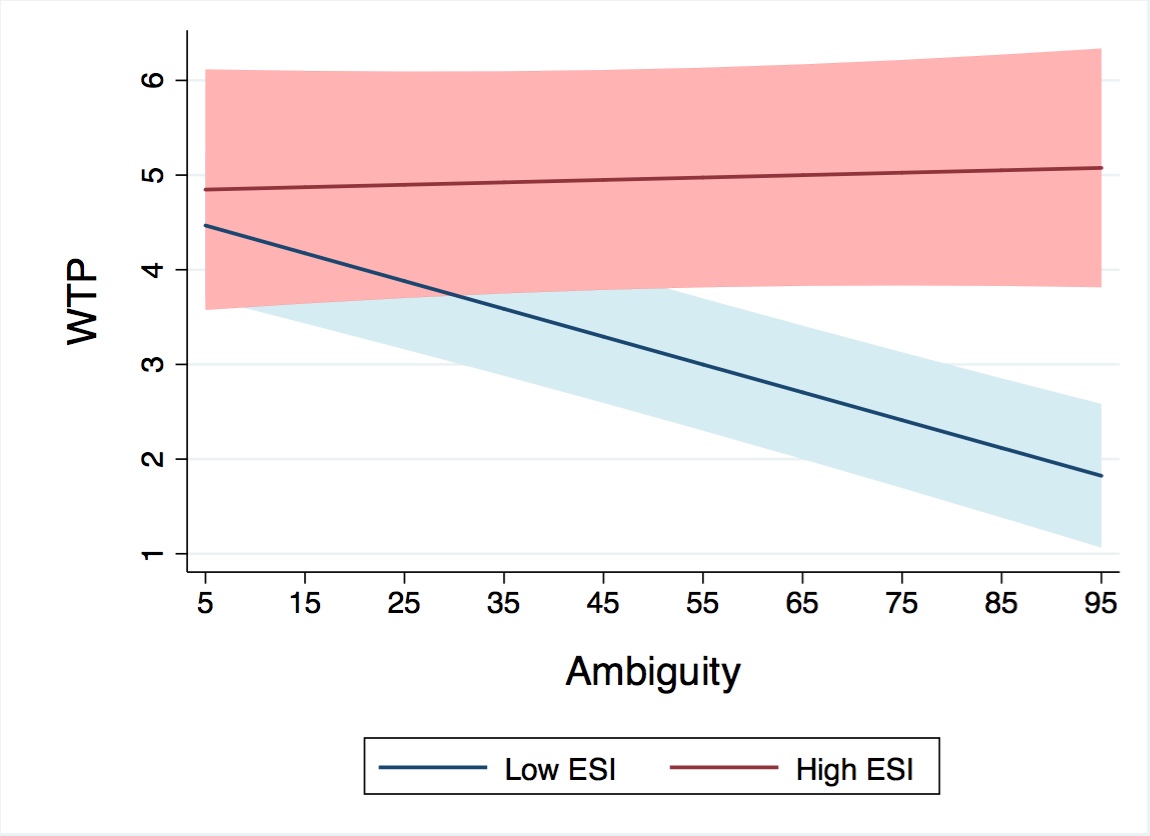


SFig 1. High Externalizing Traits Associated with Ambiguity Insensitivity.
